# An Outbreak of Institutional Dysentery Due to the Y. Bacillus

**Published:** 1915-03

**Authors:** R. H. Norgate, I. Walker Hall

**Affiliations:** Medical Superintendent, Bristol Union Workhouses; Professor of Pathology, University of Bristol


### AN OUTBREAK OF INSTITUTIONAL DYSENTERY DUE TO THE Y. BACILLUS.

BY

R. H. Norgate, M.R.C.S.,

*Medical Superintendent, Bristol Union Workhouses.*

AND

I. Walker Hall, M.D.,

*Professor of Pathology, University of Bristol.*

---

At the close of last year there was an outbreak of epidemic diarrhœa at the Stapleton Workhouse. It commenced about the middle of November, reached its height at the end of December, and gradually receded during the closing days of January. In all, some forty persons were attacked, of whom five died. The first case was that of a man, S., who had been working in connection with Colonial troops on Salisbury Plain. He was admitted to hospital ward on November 23rd, and had recovered sufficiently to be discharged on December 2nd. During this time one of the nurses attending him was transferred to a female ward. Five days later the inmates of the female ward showed signs of the same condition, and the whole of the patients and the ward staff were in turn infected. Four visitors to the ward suffered similarly. One of the nurses went to her home on a visit. Four days later her father and a relative were taken ill. One of the patients from the same ward entered a home in Clifton. Several days passed without remark, then four other servants were prostrated with dysenteric evacuations. After this there was a lull of fourteen days, succeeded in turn by a final outburst of some twelve cases.

The following brief clinical histories may be of interest :—

**Case 1.**—On November 23rd a middle-aged man (a groom), S., was admitted to the casual wards with a history of diarrhœa and blood in his stools. He had felt ill for a week. He was kept under observation, and showing no signs of improvement was transferred to a male ward in the Workhouse Infirmary on November 24th. He had been working on Salisbury Plain for some time, digging trenches, and attending to horses in the Canadian lines. He is an habitual tramp, and visits these wards about once in a month, and as a rule is clean and tidy in his appearance, but on this occasion was unkempt, and had vermin on his hair and clothing. For the first twenty-four hours he had several very loose stools with a greenish scum, and much blood and slime. He was given a large dose of castor oil, and on the third day he was much relieved and the diarrhœa was stopped. He took his discharge on December 2nd, apparently well. The night nurse in this male ward was M. On November 28th she was transferred to a ward containing about twenty-two old ladies, whose ages varied between sixty and ninety years.

**Case 2.**—On the evening of December 4th an aged woman, B., suddenly collapsed while I was in the ward. During the day she had passed five very watery stools with some blood. She revived with difficulty, and remained very ill up to December 9th, without any further loose stools or bleeding.

**Case 3.**—An aged woman, W., on December 8th was seized in the same way, and did not recover until December 10th.

**Case 4.**—On December 9th an aged woman, L. i., with a huge ventral hernia, was suddenly taken ill. Temperature 102.2° F., pulse 160, respiration 36, frequent vomiting, numerous offensive stools containing blood and mucus. I at first considered that it might be a gastric form of influenza, as there was a good deal of ordinary influenza in the hospital. During the next four days the condition continued, and the stools changed to a greenish colour. She died on December 14th.

**Case 5.**—On December 12th another aged woman, L. ii., had loose stools, offensive and bloody. The bowels were thoroughly washed out with saline solution. On the 17th she collapsed, the stools became of a dark chocolate colour, and she died on December 18th.

**Case 6.**—E., who also had a huge ventral hernia, started her attack on December 13th, and for the first three days her temperature was normal, although the stools were copious, slimy and bloody. She apparently had a second infection on the 16th, and began to vomit; her temperature rose and the motions were green. She died on December 21st.

**Cases 7 and 8.**—Nurses N. M. and N. W., who were in the ward, both had much pain and frequent stools with slight hemorrhage.

**Case 9.**—Nurse N. W. went home for a day into the Midlands to see her relations, and three days later her father had a severe attack of dysentery and was ill for several days.

**Cases 10 and 11.**—The Chaplain and his wife both visited this ward on December 20th, and spent some time with the old people, especially with E. The Chaplain's wife had a severe dysentery five days later, and the Chaplain a lesser attack in three days after that.

**Case 12.**—H., an aged woman of ninety, had diarrhœa on the 15th, with a temperature of 101.8° F. The stools contained blood on the 16th. On the 20th they showed an excess of mucus and were dark in colour, becoming subsequently definitely green. She died on December 22nd. Pulv. ipecac. gr. 60 was given twice.

**Case 13.**—The wardswoman, W. ii., aged 46, had loose stools with blood on the 17th. She had frequent lavage for six days, and she made a good recovery. Her bowels were not opened for seven days after. She had some jaundice.

**Case 14.**—A comparatively new admission, R., with a previous history of rheumatism and heart failure, had sudden loose stools on the 16th. Suppression of urine followed, and she collapsed on the 23rd.

**Case 15.**—B. ii., aged 90, became ill on December 18th, vomited a greenish yellow fluid. She was very jaundiced. Temperature 96.6° rising the next day to 100.2° F., and blood appeared in her stools. With pulv. ipecac., and frequent saline irrigations, she began to improve, and on December 28th passed a formed stool.

**Case 16.**—L. iii., a case of cerebral hemorrhage, was admitted and remained comatose for five days. She was fed with milk with the greatest difficulty. On the 19th she passed three loose and offensive blood-tinged stools. This condition continued on and off for several days. She was maniacal and noisy. After two injections of gr. $\frac{1}{3}$ emetine the diarrhœa ceased and her mental state appeared normal.

**Case 17.**—B. iii., a case of neuritis, had dysenteric stools for three days.

**Case 18.**—L. iv. had a severe dysentery for two days and then recovered.

**Case 3.**—W. had apparently a second infection on January 1st. Temperature 100° F., numerous dark stools followed by definite hemorrhage. On January 2nd a large intestinal cast (9 inches) was passed. She died on January 8th.

From December 12th special precautions were taken in this ward, and nurses and patients were isolated. The lavatories were specially disinfected, and all bedding soaked in carbolic solution 1 in 20, before being transferred to the laundry. Special instructions were issued to the nurses with regard to their hands and dresses. About this time I communicated with the Medical Officer of Health, Dr. D. S. Davies, after a negative Widal reaction had been obtained in the case of B. iii. He kindly visited the wards, and advised that bacteriological examinations should be made of the blood and stools of some of the patients. These were carried out by one of us (I. W. H.).

**Cases 19 to 26.**—On January 28th I was informed late in the evening that seven day nurses, including N. M., had been seized with abdominal pain and violent diarrhœa. They were treated with brandy and port wine, morphia and capsicum. One nurse had a temperature of 99° F., the temperatures of the others were below 97° F. They were collapsed and in great pain. I saw them once during the night, and except for one with temperature 99° F., they were all on duty the following day. This nurse had diarrhœa and abdominal pains for three days with increased temperature and dysenteric stools.

**Case 27.**—The superintendent nurse had diarrhœa and colicky pains for three days, but she had had colitis more than once before. I think her case was functional, and possibly the same remark may apply to two junior nurses who were diarrhœic the next day. All these nurses had eaten some mince pies for dinner, but others had consumed portions from the same lot and were not ill.

**Cases 30 to 31.**—On January 11th a senior official of the Institution passed ten stools in two hours, and several more sixteen hours later. The diarrhœa then ceased.

A visitor to his house was attacked similarly on January 13th.

**Case 32.**—Nurse L. had a severe attack on January 14th. She was ill for three days with typical stools.

**Case 33.**—On January 12th S., in dysentery ward, had an attack. She recovered in two days.

**Case 34.**—B. iv., who cleaned the knives and forks and silver in the kitchen of the hospital, was taken ill with dysenteric stools containing much blood.

**Case 16** had another relapse after eating currant cake, surreptitiously given her by a friend.

The Chaplain's wife had another relapse.

The other pat ents in the ward had been in ordinary health.

The same two day nurses, N. M. and N. W., have been in charge of the cases during the outbreak.

A patient, H. ii., who left this ward on January 7th, having refused to stay any longer, returned on January 26th with the following history : On leaving she went to a home for servants in Clifton. For four or five days after leaving here she was in bed for three days with acute diarrhœa. Three days later four other girls in this same house were taken ill with blood and slimy motions and severe pain. They put it down to the drinking water, although the head of the home was informed by the patient of what had occurred in Stapleton.

SCHEMA SHOWING THE VARIOUS INFECTIONS.

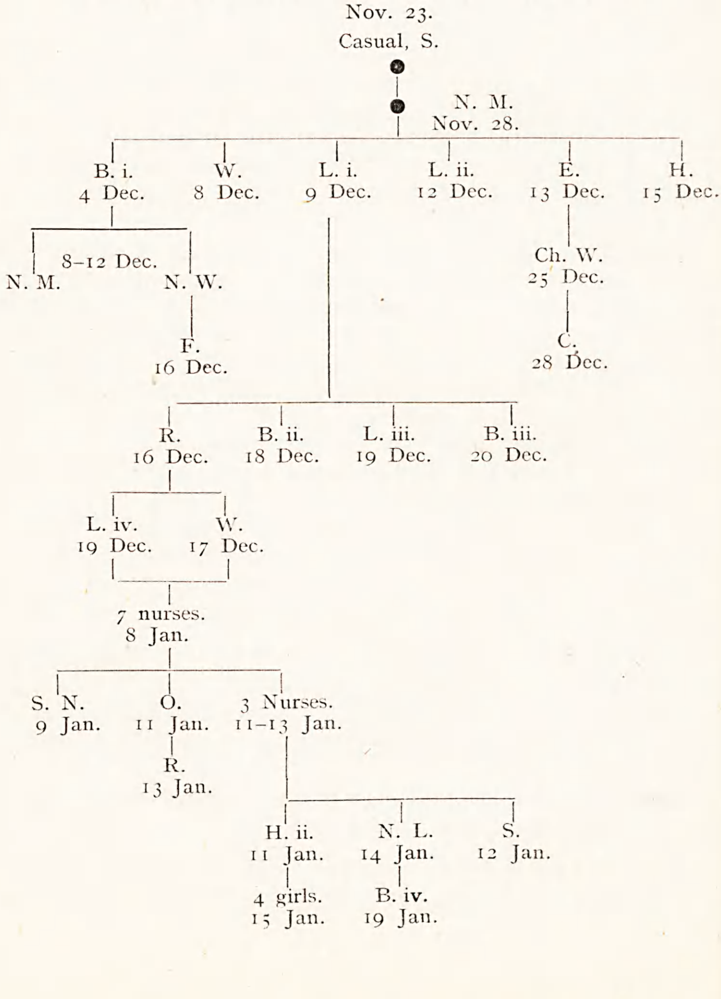

*Bacteriology.*—Bacteriological examinations were made when the outbreak was well established. After an incubation period of about four days the stools contained pus, blood and mucus for about a week in the severe cases. Amœbæ were not present.

Upon isolation (see Table No. II., Cases 1 and 2) streptococci and anærobes were not more numerous than usual. The strain of the bacillus coli present was slightly abnormal, in that it fermented lactose more slowly than average strains. Typhoid, paratyphoid, cholera and Morgan's bacilli were not present.

An organism was isolated from the mucous strands which exhibited the following characteristics :—

It grew well on ordinary media at 22° C. and 27° C., both anaërobically and aërobically. It proved to be a non-motile, non-sporing, gram-negative rod, which did not exhibit any chromatin granules. It was small and slender, and did not show pleomorphism nor any special arrangement.

On solid media it formed colonies which were at first round and translucent, and later presented irregular margins, and a creamy, non-granular centre. In streaks the film was translucent and finally creamy.

Anaërobically, in stab cultures it yielded a slightly granular growth without projections.

Gelatine was not liquefied.

It fermented glucose, lævulose and mannite, without the formation of gas. Litmus milk was turned alkaline after seventy-two hours without being clotted. Maltose, galactose, arabinose, raffinose, lactose, saccharose, sorbite, dulcite, adonite, dextrin, starch and inulin were not fermented.

It was not agglutinated by standard Shiga or Flexner serum, but was agglutinated by standard Y. serum in a dilution of 1 in 3,000. The titre of the standard was 1 to 3,200.

*Indol* was not formed in suitable peptone water.

*Nitrates* were reduced.

*Nitrites* were also reduced in accordance with the work of Logie.

*Inoculation.*—The bacillus appears to be one of low virulence. Two standard loopsful of an agar culture were injected into a guinea pig without producing any effect. One-sixth of an agar culture failed to kill another guinea pig, but induced the formation of a local abscess near to the seat of the injection. The pus present in the abscess contained a few bacilli, and permitted a feeble growth upon transference to a veal broth medium. Cultures made from the heart blood were negative. The blood of the second guinea pig agglutinated the bacillus 1 in 125, and also an organism obtained from a second specimen of fæces (Waite's), but did not agglutinate Flexner's or Shiga's bacilli. It was without effect on any of the associated organisms recovered from the other cases described in connection with the outbreak.

A white mouse survived an injection of three loopsful of an agar culture for over a week.

The morphological, cultural, biochemical and inoculation characters exhibited by the organism allow of its identification as the Y. bacillus of dysentery described by Hiss-Russell.

The blood of the patients were examined for the antibody content as follows :—

*Agglutinins.*—Serum obtained from the patients was tested against the bacillus and against coli, coliform and streptococcal organisms isolated from the fæces received, and also upon stock cultures of Shiga and Flexner dysentery bacilli. The following are the results :—

TABLE I.

| | *Agglutination.* |
|---|---|
| Bacillus isolated from stool .. .. | 1 in 125. |
| Patient's own B. coli .. .. .. | no agglutination. |
| Patient's own coliform organisms .. | do. |
| Patient's own streptococci .. .. | do. |
| Shiga's bacillus .. .. .. .. | do. |
| Flexner's bacillus .. .. .. | do. |

Absorption experiments were performed, and it was found that the whole of the agglutinins were removed by the bacillus isolated in the fæces. After the removal of these specific agglutinins, the supernatant fluid agglutinated the patient's B. coli and a stock B. coli in a dilution of 1 in 20, but was without effect on the coliform organisms, streptococci, Shiga's or Flexner's bacilli.

*Complement Deviation.*—The deviation of complement could not be obtained even with large doses of the patient's serum, when antolysed emulsions of the Y. bacillus, Flexner's bacillus, and Shiga's bacillus were used as antigens.

The organisms present in the stools from which the B. dysenteriæ could not be isolated exhibited certain features worthy of comment (see Table II.). The Pyocyaneus bacillus was present in the case of M. L.; it may have been simply an associated factor. Green pigmentation of the stools was noted in all the fatal cases. In the other cases non-motile bacteria were obtained, which gave reactions indicating their relation to the Dysentery group; none, however, were sufficiently definite to be considered as identical with the Y. bacillus.

The agglutinative powers show a correspondence with the stage of the condition and the time of the removal of the blood (see Tables I. and II.). The short period of persistence is in agreement with the slight intensity of the attack, for

TABLE II.

| CASES. | Date of attack. | FAECES. | | BLOOD. | | |
|---|---|---|---|---|---|---|
| | | | | *Agglutination.* | | |
| | | | | | Bacillus Y. | |
| | | Microscopical. | Bacterio-logical. | Own organisms other than Bacillus Y, Jan. 20, 1915. | Jan. 20, 1915. | Feb. 2, 1915. |
| 1. B. .. | Dec. 13 | Dec. 19—Pus. Mucus. R.B.C. .. .. .. | Bacillus Y | (Dec. 15.) 1/20 | 1/125 | — |
| 2. S. W. | Jan. 1 | Jan. 8—Pus. Mucus. No R.B.C. .. .. | Bacillus Y .. | 1/20 | 1/50 | — |
| 3. M. L. | Dec. 19 | Jan. 10—No pus. No mucus. No R.B.C. .. | No Bacillus Y | 0 | 0 | 0 |
| 4. L. .. | Jan. 14 | Jan. 23—No pus. No mucus. No R.B.C. .. | No Bacillus Y | 0 | 0 | 0 |
| 5. E. B. | Jan. 19 | Jan. 23—No pus. No mucus. No R.B.C. .. | No Bacillus Y | 0 | 0 | 0 |
| 6. S. .. | Jan. 12 | Jan. 23—No pus. No mucus. No R.B.C. .. | No Bacillus Y | 0 | — | 0 |
| 7. W. | Dec. 17 | Jan. 23—No pus. No mucus. No R.B.C. .. | No Bacillus Y | 0 | 1/50 | 0 |
| 8. B. | Dec. 20 | Jan. 23—No pus. No mucus. No R.B.C. .. | No Bacillus Y | 0 | 1/125 | 0 |
| 9. M. | Nov. 28 | Feb. 16—No pus. No mucus. No R.B.C. .. | No Bacillus Y | — | 1/50 | — |
| 10. W. | Dec. 8 | Feb. 16—No pus. No mucus. No R.B.C. .. | — | — | — | — |

TABLE III.

OTHER BLOOD EXAMINATIONS.

| Cases. | Attack Date. | *Agglutination*, Feb. 1st, 1915. | | |
|---|---|---|---|---|
| | | Bacillus Y. | Bacillus Dysenteriæ (Shiga). | Flexner. |
| B. .. .. .. | Jan. 8 | 0 | 0 | 0 |
| J. .. .. .. | Jan. 8 | 0 | 0 | 0 |
| G. .. .. .. | Dec. 20 | 0 | 0 | 0 |
| N. M. .. .. .. | Dec. 20 | 0 | 0 | 0 |
| N. W. .. .. .. | Dec. 20 | 0 | 0 | 0 |
| B. i. .. .. .. | Dec. 4 | 0 | 0 | 0 |
| O. .. .. .. | Jan. 11 | 0 | 0 | 0 |

although the attacks proved fatal to a few old people, those previously in good health showed no permanent after-effects (see Tables II. and III.).

From a consideration of the clinical histories, the low virulence of the organism concerned and the agglutinative reactions obtained in those cases in which the Y. bacillus was not demonstrable in the stools, it is permissible, perhaps, to assume that this Y. organism was the chief factor in the causation and spread of the infection. The fact that in more acute cases the dysentery bacillus may disappear at an early stage of the condition and be replaced by local organisms, strengthens the supposition.

Unfortunately, owing to his renewal of tramping, it was impossible to obtain for examination a specimen of the fæces of the supposed original introducer of the organism to the workhouse precincts. However, on March 24th he passed through Bristol, and the opportunity was taken to determine his agglutination titre for the Y. bacillus. It was found to be 1 in 20.

It will be noted that there was an interval in the attacks extending from December 20th to January 8th. In order to determine the cause for the recrudescence of the attacks in January, and with a view to eliminate the possibility of any further transmission, the stools of intermediary "carriers" and of those recovered from the infection have been examined (see Table II., 3 to 10). The Y. bacillus has not been obtained from any of them.

### Progress of the Medical Sciences.

